# Anti-CD19 CAR-T as a feasible and safe treatment against central nervous system leukemia after intrathecal chemotherapy in adults with relapsed or refractory B-ALL

**DOI:** 10.1038/s41375-019-0437-5

**Published:** 2019-03-07

**Authors:** Xiaoyuan He, Xia Xiao, Qing Li, Yanyu Jiang, Yaqing Cao, Rui Sun, Xin Jin, Ting Yuan, Juanxia Meng, Li Ma, Wenyi Lu, Cuicui Lyu, Kaiqi Liu, Mingfeng Zhao

**Affiliations:** 1 Nankai University School of Medicine, Tianjin, PR China; 2 Department of hematology, Tianjin First Central Hospital, No. 24 Fu Kang Road, Tianjin, PR China; 3 Tianjin medical university, Tianjin, PR China; 4 Leukemia Center, Institute of Hematology and Blood Diseases Hospital, Chinese Academy of Medical Sciences and Peking Union Medical College, 288 Nanjing Road, Tianjin, PR China

**Keywords:** Acute lymphocytic leukaemia, Immunotherapy

Chimeric antigen receptor (CAR)-T cell treatment as an emerging tumor immunotherapy has produced exciting results in relapsed or refractory B cell acute lymphoblastic leukemia (B-ALL) [1–4]. However, few data are available on the therapeutic effect of CAR-T against central nervous system leukemia (CNSL). Here, we evaluated the feasibility and safety of anti-CD19 CAR-T against CNSL after intrathecal chemotherapy in three adults with relapsed or refractory B-ALL.

Patient 1 with isolated CNSL, was refractory to high dose methotrexate plus vindesine and L-asparaginase, and intrathecal chemotherapy, accompanied by bone marrow (BM) sustained remission with minimal residual disease (MRD) negative. Patient 2 initially experienced a CNS relapse, and underwent intrathecal chemotherapy, systemic chemotherapy and radiotherapy. However, her CNSL was not controlled, accompanied by BM recurrence. Patient 3 received prophylactic intrathecal chemotherapy after his first complete remission (CR) but experienced a rapid recurrence in his BM and CNS. They were enrolled in our anti-CD19 CAR-T clinical trial (ChiCTR-ONN-16009862). Prior to CAR-T cell infusion, all the patients received conditioning chemotherapy with fludarabine and cyclophosphamide, and intrathecal chemotherapy to reduce blasts in the cerebrospinal fluid (CSF). Detailed patient and methodological information are described in Supplementary Methods, Table S1, and Figure S1.

First, we assessed the clinical response of CNSL to CAR-T therapy in these patients. All patients with CNSL achieved CR approximately one to two weeks post CAR-T infusion (Fig. 1a–c), accompanied by BM remission with MRD negative in patient 2 and patient 3 (Fig. 1d). One month after CR, patient 2 received allogeneic hematopoietic stem cell transplantation. Until the most recent follow-up, her leukemia free survival has been over 2 months. Interestingly, patient 1 and patient 3 receiving no further therapy for CNSL after CAR-T infusion were in sustained remission for over 5 months (Table S1). A phase 1 dose-escalation trial reported that two B-ALL patients with CNSL achieved CR after CAR-T therapy [3]. Another clinical trial showed that two CNSL patients at the time of CAR-T infusion subsequently had no blasts in the CSF [2]. Dai and colleagues also reported that two patients with active CNSL at the time of CAR-T infusion became CNS negative [5]. Altogether, CAR-T can be a feasible and effective treatment for CNSL.

**Fig. 1:**
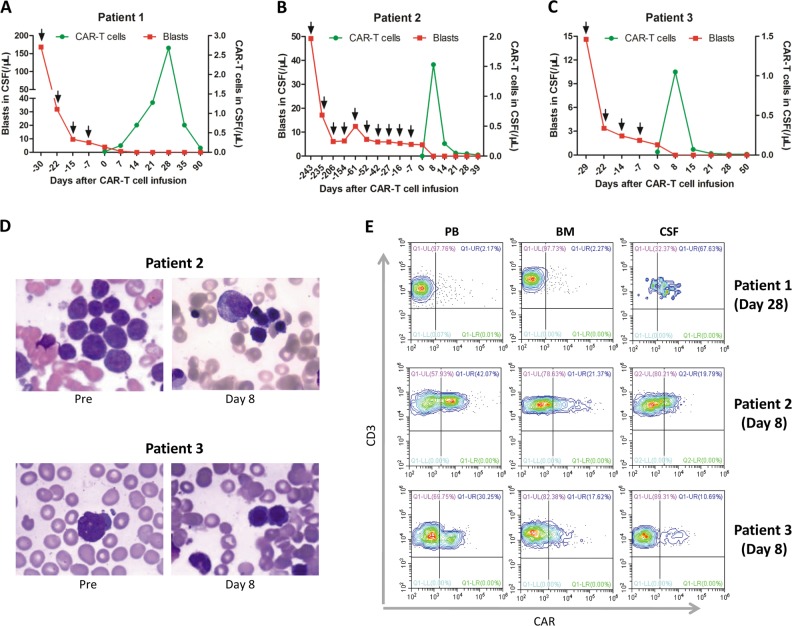
Clinical response, expansion and persistence of anti-CD19chimeric antigen receptor (CAR)-T cells. **a**–**c** Cell counts of CAR-T cells and tumor cells in the cerebrospinal fluid (CSF) before and after CAR-T infusion. The black arrows indicate intrathecal chemotherapy. **d** Malignant cells in the bone marrow (BM) of patient 2 and patient 3 pre and post-infusion. **e** The peak expansion levels of CAR-T cells in the peripheral blood (PB), BM, and CSF

We further evaluated the proliferation and persistence of CAR-T in vivo. Two patients reached the peak expansion of CAR-T cells in the CSF on day 8 (Fig. 1b, c), which coincided with the disappearance of CSF blasts in the responding patients. However, patient 1 exhibited peak expansion on day 28, 2 weeks later than the disappearance of the tumor cells in the CSF (Fig. 1a). The persistence time of CAR-T cells in the CSF of patient 2 and patient 3 was about 2–3 weeks (Fig. 1b, c), while in patient 1, 5.19% of CAR-T cells persisted in the CSF on day 90 (Fig. 1a). Patient 1 who only had CNS relapse, showed significantly higher peak proportions of CAR-T cells in the CSF than those in the peripheral blood (PB) and BM. However, patient 2 and patient3 both with BM and CNS recurrence exhibited markedly lower peak levels of CAR-T cells in the CSF compared to patient 1 (Fig. 1e), but relatively high peak levels in the PB and BM. These different distributions of CAR-T cells may be explained by chemotaxis and stimulated proliferation of effector cells at the tumor sites.

We next evaluated the adverse events associated with anti-CD19 CAR-T treatment. Patient 1 only showed grade 1 anemia and grade 4 lymphopenia on day 3 after infusion (Table S2). She didn’t complain of any discomfort post CAR-T therapy. It was reported that the severity of the cytokine release syndrome (CRS) was correlated with tumor burdens and T cell proliferation [2, 3, 6]. However, the peak expansion time of patient 1 was two weeks later than the disappearance of the tumor cells in the CSF (Fig. 1a), which may explain the low risk of CRS. Patient 2 and patient 3 experienced grade 2 fever, grade 3 febrile neutropenia, grade 2 CRS, and grade 1 reduced consciousness. Patient 2 also had grade 1 cognitive impairment and grade 2 convulsion. Dexamethasone was administrated at 10 mg q8h to control her seizure on day 8, and was de-escalated on day 9 and discontinued on day 10. These adverse events were well managed with supportive care and dexamethasone. Other adverse events related to CAR-T therapy are shown in Figure S2 and Table S2.

In summary, this study showed that anti-CD19 CAR-T could effectively eliminated leukemia cells in the CNS with fully reversible toxicity. We also found that patient with only CNS recurrence had higher levels of CAR-T in the CSF and relatively lower severity of toxic effects than those with BM and CNS recurrence. This study shows that anti-CD19 CAR-T might be a feasible and safe treatment for CNSL after intrathecal chemotherapy in adults with B-ALL, especially in isolated CNSL. More cases and further studies are needed to verify these findings.

## Supplementary information

Supplementary Information
