# Supplementary material for: Anti-CD19 CAR-T as a feasible and safe treatment against central nervous system leukemia after intrathecal chemotherapy in adults with relapsed or refractory B-ALL
Source: Leukemia. 2019 Mar 7;33(8):2102–4. doi: 10.1038/s41375-019-0437-5 (PMC6756216; doi:10.1038/s41375-019-0437-5)
Supplement: Supplementary file 1 — Supplementary Information [file 41375_2019_437_MOESM1_ESM.docx]

**Correspondence**

**Anti-CD19 CAR-T as a feasible and safe treatment against central nervous system leukemia after intrathecal chemotherapy in adults with relapsed or refractory B-ALL**

Running title: Chimeric antigen receptor-T therapy might be feasible and safe for central nervous system leukemia

Xiaoyuan He^1^, Xia Xiao^2^, Qing Li^2^, Yanyu Jiang^2^, Yaqing Cao^3^, Rui Sun^3^, Xin Jin^1^, Ting Yuan^2^, Juanxia Meng^2^, Li Ma^2^, Wenyi Lu^2^, Cuicui Lyu^2^, Kaiqi Liu^4,*^, Mingfeng Zhao^2,1,*^

^1^Nankai University School of Medicine, Tianjin, PR China

^2^Department of hematology, Tianjin First Central Hospital, Tianjin, PR China

^3^Tianjin medical university, Tianjin, PR China

^4^Institute of Hematology and Blood Diseases Hospital, Tianjin, PR China

*These authors contributed equally to this work.

*Corresponding author: Mingfeng Zhao, MD&PhD. Department of Hematology, Tianjin First Central Hospital, Nankai University School of Medicine, No. 24 Fu Kang Road, Tianjin, 300192, China. Tel.: +86-22-2362-6946. Fax: +86-22-2362-6492. E-mail: mingfengzhao@sina.com. Kaiqi Liu, MD. Leukemia Center, Institute of Hematology and Blood Diseases Hospital, Chinese Academy of Medical Sciences and Peking Union Medical College, 288 Nanjing Road Tianjin, 300020, China. Tel.: +86-13602127632. Fax: +86-22-23909020. E-mail: yanghua94583@sina.com.

Conflict of interest: The authors declare no potential conflicts of interest.

**Supplementary Information**

**Supplementary Materials and Methods**

**Study design and patient characteristics**

As of November 20, 2018, three CD19^+^ B-ALL patients (two female, one male) with CNSL aged 20-43 years have been enrolled in our CAR-T clinical trial for relapsed or refractory ALL. There was one isolated CNSL patient, and two with BM and CNS involvement. Prior to CAR-T cell infusion, all patients received conditioning chemotherapy with fludarabine (30mg/m^2^/day, day -4 to day -2) and cyclophosphamide (750mg/m^2^/day, day -2). And all of them were treated with intrathecal chemotherapy to reduce blast cells in the cerebrospinal fluid (CSF) before CAR-T infusion except day 0. This study was conducted according to the principles of the Declaration of Helsinki and with the approval of the Ethics Committee of Tianjin First Central Hospital. All the enrolled patients or their families provided written informed consent. Additional details of patient characteristics are described in Table S1.

**Preparation of anti-CD19 CAR-T cells**

At the beginning of CAR-T cell manufacturing, all the patients should undergo leukapheresis to obtain peripheral blood mononuclear cells. Then the leukapheresis products were sorted for CD3 positive T cells by magnetic beads coated with CD3 antibody (#130-097-043, [Miltenyi Biotec](https://www.miltenyibiotec.com/), Germany). Interleukin 2 and paramagnetic beads coated (11131D, Thermo Fisher Scientific, USA) with antibodies to CD3 and CD28 were added to stimulate the expansion of T cells. In the current study, T cells were transduced with lentivirus to generate CAR-T cells. Generally, these T cells were engineered via 10-12 days manufacturing process to express a CD19-CAR incorporating an anti-CD19 single-chain variable fragment plus TCR zeta and CD28 signalling domains. CAR-T cells were released once they met the criteria for infusion. The percentage of CAR transduction in the three patients was approximately 40% to 70%.

**Clinical response assessment for CNSL**

The tumor burdens in the CSF were measured by means of flow cytometry before and after CAR-T cell treatment. Clinical response assessment for CNSL was done every seven days (within 1 day) in the first month post anti-CD19 CAR-T infusion. The patients in whom blast cells were not detectable in the CSF, accompanied by the disappearance of clinical symptoms and normal cerebrospinal fluid examination results were considered complete remission (CR).

**Evaluation of CAR-T cell persistence and expansion in the CSF**

The proportions of CAR-T cells in CD3 positive T lymphocytes and the cell counts of CAR-T cells in the CSF were detected via flow cytometry before (day 0) and after CAR-T cell infusion. The time points for evaluation of CAR-T proliferation and persistence were at the same day with tumor cells detection in the CSF, peripheral blood and bone marrow.

**Assessment of toxic effects after CAR-T infusion**

Toxicities were evaluated relative to a baseline assessment conducted before CAR-T cell infusion. The concentrations of serum inflammatory markers including IL-2R, IL-6, IL-8, IL-10, TNF-α, IFN-γ, ferritin and C-reactive protein (CRP) were evaluated by Luminex assay, according to the manufacturer’s instructions.

**Supplementary Figures**


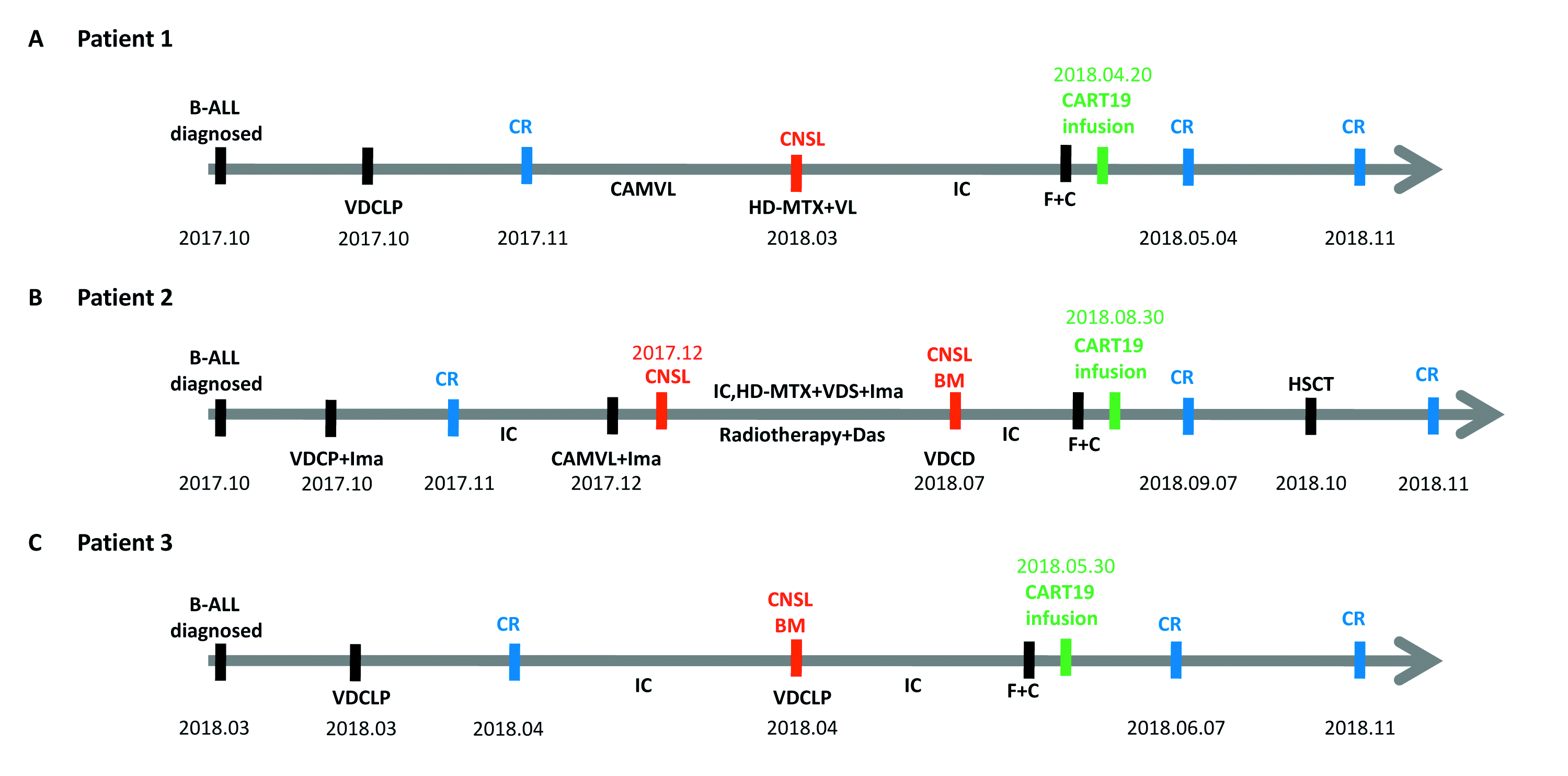


**Supplementary Figure 1. The treatment courses before chimeric antigen receptor (CAR)-T cell therapy.** The treatment courses in patient 1 (A), patient 2 (B), and patient 3 (C).VDCP, vindesine, daunorubicin, cyclophosphamide, and prednisone. EA, etoposide and cytarabine. HD-MTX, high dose meuthotrexate. IC, intrathecal chemotherapy. CR, complete remission. CNSL, central nervous system leukemia. Ima, imatinib. F+C, fludarabine and cyclophosphamide. VDCLP, vindesine, daunorubicin, cyclophosphamide, prednisone, and L-asparaginase. CAMVL, cyclophosphamide, cytarabine, mercaptopurine, vindesine, and L-asparaginase. VL, vindesine and L-asparaginase. VDS, vindesine. Das, dasatinib. HSCT, haematopoietic stem cell transplant.


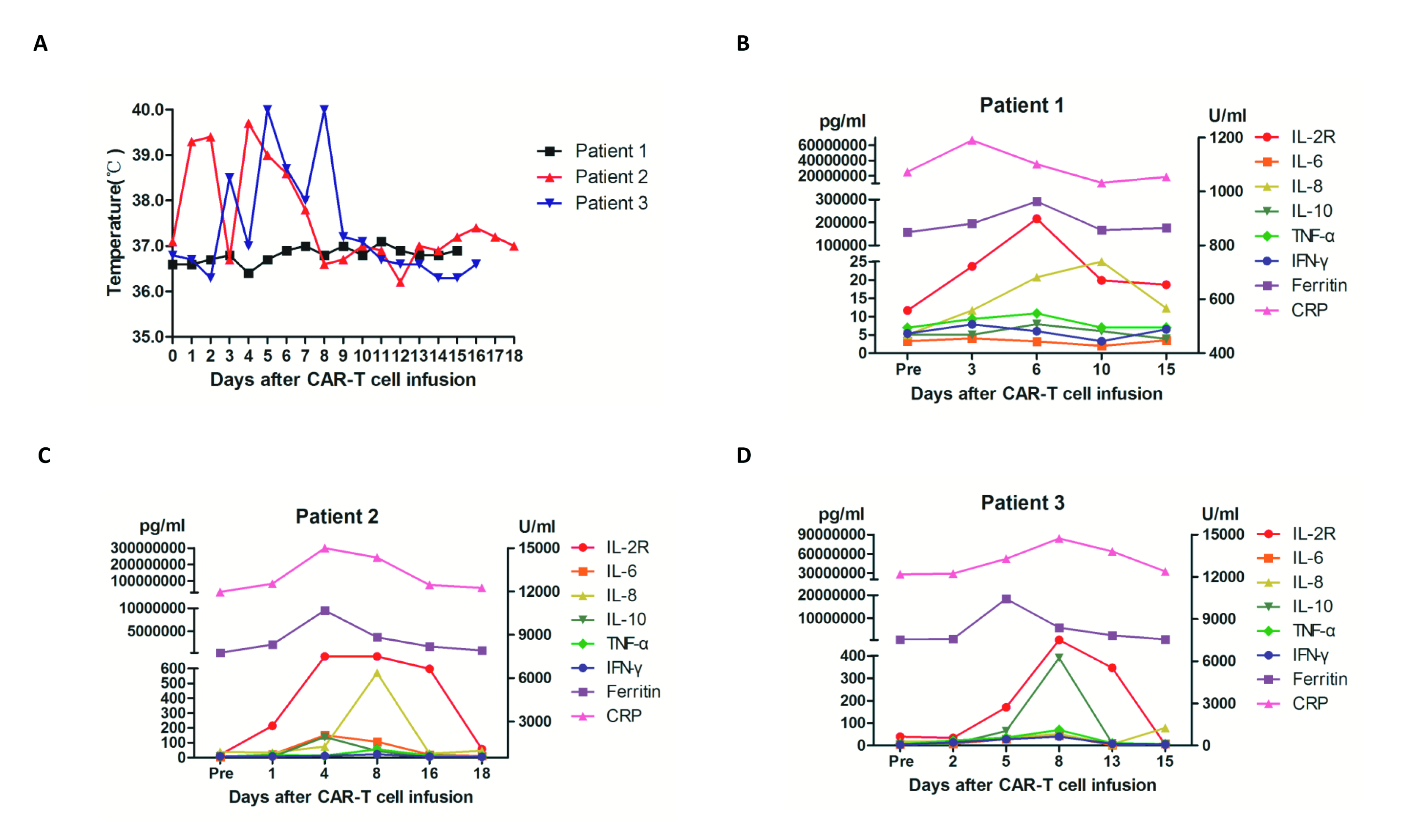


**Supplementary Figure 2. Changes of body temperature and inflammatory levels after anti-CD19 chimeric antigen receptor (CAR)-T cell treatment.** (A) Body temperature fluctuation monitoring. The levels of inflammatory markers in patient 1 (B), patient 2 (C), and patient 3 (D). IL-2R is indicated on the right Y-axis and the other markers are shown on the left Y-axis. CRP, C-reactive protein.

**Supplementary Tables**

**Supplementary Table 1. The clinical characteristics of B-ALL patients with central nervous system leukemia**

| Patient no./ Gender/Age | Diagnosis/  Molecular biology | CNS status | Extramedullary  involvement | Total dose of CAR-T cells/kg | CNSL response to CAR-T | CRS grade | Leukemia free survival (months) | Bridge  to  HSCT |
| --- | --- | --- | --- | --- | --- | --- | --- | --- |
| 1/F/34 | B-ALL/  *E2X-PBX1*^+^ | 3 | CNS | 4.4х10^6^ | CR | 0 | 6+ | No |
| 2/F/20 | B-ALL/  *EBF1-PDGFRB*^+^ | 3 | CNS | 4.2х10^6^ | CR | 2 | 2+ | Yes |
| 3/M/43 | B-ALL/  *MLL-AF4*^+^ | 3 | CNS | 6.0х10^6^ | CR | 2 | 5+ | No |

M, male. F, female. CNS, central nervous system. CNSL, central nervous system leukemia. CR, complete remission. CRS, cytokine release syndrome. HSCT, haematopoietic stem cell transplant.

**Supplementary Table 2. Observed adverse events related to chimeric antigen receptor (CAR)-T cell therapy**

| **Adverse event** | **Grade 1** | **Grade 2** | **Grade 3** | **Grade 4** |
| --- | --- | --- | --- | --- |
| **Inflammation-related event** |  |  |  |  |
| Fever |  | 2 |  |  |
| Febrile neutropenia |  |  | 2 |  |
| Cytokine release syndrome |  | 2 |  |  |
| **Haematological event** |  |  |  |  |
| Anemia | 1 |  | 2 |  |
| Leukopenia |  |  | 1 |  |
| Neutropenia |  |  | 1 |  |
| Lymphopenia |  |  | 2 | 1 |
| Thrombocytopenia |  |  | 1 |  |
| Fibrinogen decreased |  |  | 2 |  |
| **Nervous system event** |  |  |  |  |
| Convulsion |  | 1 |  |  |
| Cognitive impairment | 1 |  |  |  |
| Reduced consciousness | 2 |  |  |  |
| **Chemical laboratory abnormalities** |  |  |  |  |
| Alanine aminotransferase increased | 2 |  |  |  |
| Aspartate aminotransferase increased | 1 |  | 1 |  |
| Gamma glutamyl transpeptidase increased |  |  | 2 |  |
| Blood bilirubin increased |  |  | 1 |  |
| Serum creatinine increased |  | 1 |  |  |
| Brain natriuretic peptide increased |  | 1 |  |  |

Grading according to the Common Terminology Criteria for Adverse Events version 4.0 (CTCAEv4).
